# Reverse epithelial-mesenchymal transition contributes to the regain of drug sensitivity in tyrosine kinase inhibitor-resistant non-small cell lung cancer cells

**DOI:** 10.1371/journal.pone.0180383

**Published:** 2017-07-06

**Authors:** An-Fu Lee, Man-Chin Chen, Chao-Ju Chen, Chih-Jen Yang, Ming-Shyang Huang, Yu-Peng Liu

**Affiliations:** 1 School of Medicine, College of Medicine, Kaohsiung Medical University, Kaohsiung, Taiwan; 2 Division of Pulmonary and Critical Care Medicine, Department of Internal Medicine, Kaohsiung Medical University Hospital, Kaohsiung Medical University, Kaohsiung, Taiwan; 3 Graduate Institute of Medicine, Kaohsiung Medical University, Kaohsiung, Taiwan; 4 Department of Laboratory Medicine, Kaohsiung Medical University Hospital, Kaohsiung Medical University, Kaohsiung, Taiwan; 5 Department of Internal Medicine, School of Medicine, College of Medicine, Kaohsiung Medical University, Kaohsiung, Taiwan; 6 Department of Internal Medicine, Kaohsiung Municipal Ta-Tung Hospital, Kaohsiung Medical University, Kaohsiung, Taiwan; 7 Division of Geriatrics and Gerontology, Department of Internal Medicine, Kaohsiung Medical University Hospital, Kaohsiung Medical University, Kaohsiung, Taiwan; 8 Graduate Institute of Clinical Medicine, Kaohsiung Medical University, Kaohsiung, Taiwan; 9 Center for Infectious Disease and Cancer Research, Kaohsiung Medical University, Kaohsiung, Taiwan; 10 Department of Medical Research, Kaohsiung Medical University Hospital, Kaohsiung Medical University, Kaohsiung, Taiwan; University of South Alabama Mitchell Cancer Institute, UNITED STATES

## Abstract

Tyrosine kinase inhibitors (TKIs) are currently the first-line treatment for non-small cell lung cancer (NSCLC) patients with epidermal growth factor receptor (EGFR) mutations. These patients receive platinum-based chemotherapy as the second-line treatment after they develop resistance to TKIs. Many patients regain sensitivity to the TKIs used in the first-line treatment after the failure of chemotherapy. However, the molecular mechanism for the regain of TKI sensitivity is largely unknown. In this study, we established gefitinib-resistant PC9 and HCC827 cell lines, which did not harbor the EGFR T790M mutation and *MET* amplification but exhibited the epithelial-mesenchymal transition (EMT) phenotype. Overexpression of EMT inducers, Snail or Slug, in the parental lines promoted their resistance to gefitinib. The gefitinib-resistant cell lines regained their sensitivity to gefitinib and displayed reverse EMT phenotypes after long-term culture in gefitinib-free culture medium. Blockage of reverse EMT by stable expression of Snail or Slug prevented the regain of TKI sensitivity. In conclusion, reverse EMT is one of the major mechanisms for the regain of TKI sensitivity in TKI-resistant NSCLC cells, suggesting that the development of small molecules targeting the EMT process may prolong the efficacy of TKIs in NSCLC patients with EGFR mutations.

## Introduction

Approximately 50% of non-small cell lung cancer (NSCLC) patients with epidermal growth factor receptor (EGFR) mutations, such as exon 19 deletion and L858R point mutation, receive tyrosine-kinase inhibitors (TKIs) as their first-line treatment. Despite the initial response to TKI therapy, the tumor eventually recurs due to acquired drug resistance to the TKIs. In 50% of these patients, the resistance against TKIs can be explained by secondary EGFR mutations, mainly T790M point mutation. A third-generation TKI, osimertinib, targeting the EGFR T790M mutation has been recently developed and prolongs disease-free survival of NSCLC patients [1].

Although osimertinib is very effective in lung cancer patients with the secondary EGFR mutation, the other 50% of patients who do not develop the T790M mutation receive platinum-based chemotherapy as their second-line treatment, and the prognosis of these patients is poor [2]. Interestingly, rechallenge with TKIs after the failure of first-line TKI treatment and second-line chemotherapy, referred to as the “TKI holiday”, can improve the survival of patients [3–6]. This resensitization of the tumor against TKIs also occurs in some patients with the T790M mutation, suggesting other mechanisms may play a role in this response [6, 7]. Multiple mechanisms of acquisition of TKI resistance other than the emergence of the T790M mutation have been explored, including transformation to small-cell lung cancer, *human epidermal growth factor receptor 2* (*HER2*) amplification, and activation of secondary pathways, such as MET-mediated pathway [8]. However, the association between these mechanisms and the regain of TKI sensitivity is largely known.

Epithelial-to-mesenchymal transition (EMT) is a reversible process that controls the phenotypic changes due to cell mobility [9], metabolic reprogramming [10], acquisition of stemness [11], and drug responses [12]. In addition to morphological changes, cells undergoing EMT are characterized by molecular alterations of junctional and cytoskeleton proteins and transcriptional regulators [13]. Under physiological and pathological conditions, EMT can be induced by extrinsic factors, such as growth factors and cytokines, or intrinsic regulators, such as Twist1, Snail and Slug. Experimental evidence has shown that the removal of EMT inducers results in the reversal of EMT and EMT-associated phenotypes [14], and a significant proportion of cancer cells exhibit intermediate states of EMT or hybrid EMT [15]. Moreover, an increasing number of studies have indicated that EMT plasticity is critical for the physiology of the cancer cells during the progression of malignancy [16, 17].

EMT has long been linked to resistance of lung cancer cells to chemotherapy [18, 19] and TKI treatment [20, 21]. Loss of E-cadherin, an epithelial marker, sensitizes lung cancer cells with primary EGFR mutations to TKI treatments and is correlated with the poor prognosis of lung cancer patients [22]. Since EMT is a dynamic process during the course of therapy, it is necessary to understand whether EMT and reverse EMT contribute to the regain of TKI sensitivity in lung cancer patients. In this study, EMT was observed in gefitinib-resistant NSCLC cell lines that were established by a stepwise escalation of gefitinib. Long-term culture of the resistant cells in gefitinib-free medium resulted in the reversal of EMT, and the cells regained their sensitivity to gefitinib. Maintenance of the EMT status by introducing constitutive expression of Snail preserved gefitinib resistance in the cancer cells, suggesting that reverse EMT is one of the important mechanisms occurring during TKI holiday.

## Materials and methods

### Cell culture and establishment of gefitinib-resistant sublines

The human non-small cell lung cancer cell lines PC9 and HCC827, carrying EGFR exon19del E746-A750, were purchased from American Type Culture Collection. Both cell lines were maintained in RPMI-1640 medium supplemented with 10% fetal bovine serum, 100 U/mL penicillin and 100 μg/mL streptomycin. The cell lines were kept in a humidified incubator containing 5% CO_2_ at 37°C. For long-term culture, all the cell lines were tested for mycoplasma every month. The gefitinib-resistant sublines were established by stepwise escalation of gefitinib concentrations from 0.5 μM to 10 μM. Gefitinib was purchased from LC Laboratories (Woburn, MA, USA). The culture medium was replaced every 3 days. After 6 months, the resistant cells were regularly maintained in culture medium containing 10 μM gefitinib. For gefitinib-withdrawal experiments, the doubling time for the PC9-WGR and HCC827-WGR cells was determined, approximately 36.8 hours and 55.7 hours, respectively. During the gefitinib withdrawal, the PC9-WGR and HCC827-WGR cells were passaged every 3 days, and the cells were seeded at 30–40% confluency.

### Cytotoxicity assay

To determine the cytotoxic effect of gefitinib in sublines of PC9 and HCC827, cells at the density of 5×10^3^ cells/well were seeded in 96-well microtiter plates. Different concentrations of gefitinib were added 24 hours after seeding. After 72 hours of incubation, CellTiter^®^ 96 non-radioactive cell proliferation assay (Promega, Madison, WI, USA) was performed according to the manufacturer’s instructions, and the optical density was determined at 560 nm by using a microtiter plate reader (BioTek Instruments, Inc., Winooski, VT, USA). The mean absorbance at each drug concentration was normalized to the control. The IC_50_ was defined as the drug concentration that caused a 50% reduction in relative absorbance.

### Plasmid construction, shRNA and lentivirus preparation

For generation of the gene expression lentiviral plasmids, synthesized DNA fragments of *Slug* (807 bp; NM_003068.4), *Snail* (795 bp; NM_005985.3) and *Twist1* (609 bp; NM_000474.3) (Thermo Fisher Scientific, Waltham, MA, USA) were cloned into the pLex-MCS lentiviral vector. The shRNA-containing lentiviral vectors were provided by the National RNAi Core Facility, Academia Sinica, Taiwan. The identifier (ID) numbers of the two shRNA clones used for E-cadherin knockdown are as follows: TRCN0000130433 (shE-cad #1), TRCN0000131097 (shE-cad #2). The lentiviral particles for all expression plasmids and shRNAs were prepared by co-transfection with SPAX2 and pMD2G plasmids into HEK293T cells. Stable clones of individual infected cell lines were established by using puromycin (1–3 μg/mL).

### Quantitative real-time PCR

Total RNA isolation and reverse transcription were conducted using the method described previously [23]. Quantitative PCR (qPCR) was set up using Fast SYBR Green Master Mix (Thermo Fisher Scientific) and run on a Step-One Plus real-time PCR system (Thermo Fisher Scientific). Polymerase chain reaction for each gene was performed as follows: 20 seconds at 95°C followed by 40 cycles at 95°C for 3 seconds and annealing at 60°C for 30 seconds. The results were normalized to those for the housekeeping gene glyceraldehyde 3-phosphate dehydrogenase (GAPDH).

### Western blot

For Western blot analysis, the cells were harvested and lysed in 1X RIPA buffer containing protease inhibitors and phosphatase inhibitors (Roche, Mannheim, Germany). Protein concentration was determined using a Bio-Rad DC protein assay kit (Bio-Rad, Hercules, CA, USA). Total protein (20 μg) was loaded onto a 10% SDS-polyacrylamide gel for electrophoresis. Specific antibodies against E-cadherin (GTX100443, GeneTex, Irvine, CA, USA), N-cadherin (ab12221, Abcam, Cambridge, USA), Vimentin (ab92547, Abcam), Snail (#3879, Cell Signaling Technology, Inc., Danvers, MA), Slug (GTX30813, GeneTex), and Twist1 (GTX100621, GeneTex) were applied to detect the targets. Monoclonal anti-Actin antibody (Sigma, St. Louis, MO, USA) was used as the loading control. The following secondary antibodies were used: goat anti-rabbit or anti-mouse immunoglobulin G (IgG)-conjugated horseradish peroxidase (Santa Cruz Biotechnology, Santa Cruz, CA, USA). To detect the specific signals, the membranes were examined using the ECL Prime Western Blotting Detection System (GE Healthcare, Waukesha, WI, USA).

### Statistical analysis

For the quantitative real-time PCR and cell viability assay, three independent experiments were conducted, and three technical replicates for each sample in every independent experiment were performed. For all Western blot analysis, three independent experiments (cell lysate from different passages of the cell cultures) were performed. The quantitative data are presented as the mean±SD. Two-sided Student’s T-test was performed. *p*<0.05 was considered significant. Statistical analyses were performed using SPSS (Statistical Package for the Social Sciences, version 14.0) software.

## Results

### Epithelial-to-mesenchymal transition occurs in gefitinib-resistant lung cancer cell lines

**Fig 1:**
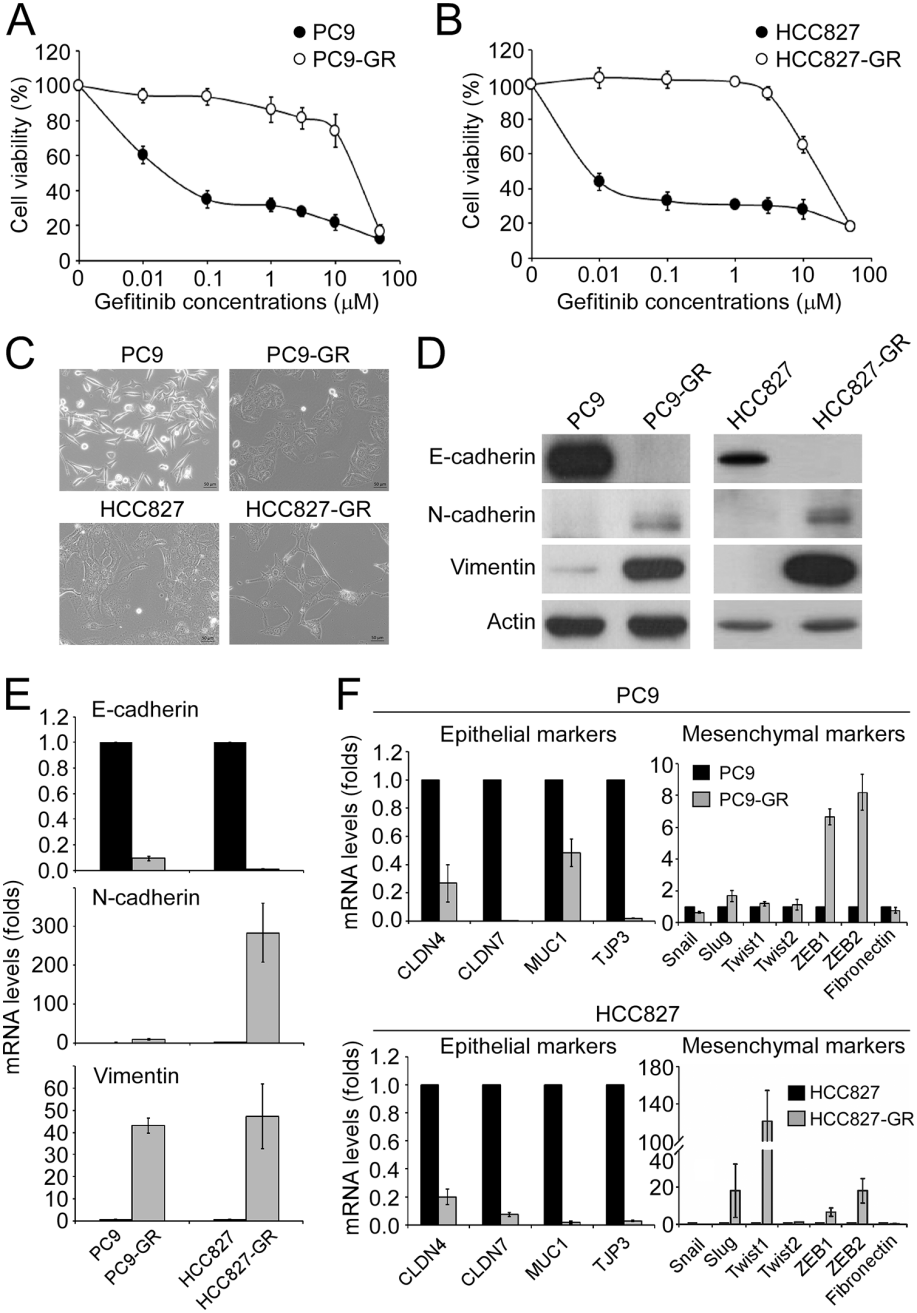
The drug-resistant PC9 and HCC827 cells underwent EMT. (A) Parental PC9 and gefitinib-resistant PC9 (PC9-GR) cells were treated with gefitinib at the indicated concentrations for 72 hours, and the relative cell viability was determined by MTT assay. (B) Viability of HCC827 and gefitinib-resistant HCC827 cells. (C) Bright-field microscopy images of parental PC9/HCC827 and gefitinib-resistant PC9/HCC827 cells. (D) Protein levels of the indicated EMT biomarkers and the internal control actin was determined by Western blot. (E) mRNA levels of E-cadherin, N-cadherin and Vimentin in parental PC9/HCC827 and gefitinib-resistant PC9/HCC827 cells were determined by qPCR. (F) qPCR analysis for the indicated epithelial (left panels) and mesenchymal (right panels) biomarkers in parental PC9/HCC827 and gefitinib-resistant PC9/HCC827 cells.

The gefitinib-resistant sublines PC9-GR and HCC827-GR were generated from their corresponding TKI-sensitive cell lines PC9 and HCC827, respectively, by following a stepwise escalation method [24]. The half maximal inhibitory concentration (IC_50_) of gefitinib for PC9-GR (24.0±7.2 μM) and HCC827-GR (25.7±3.2 μM) sublines were dramatically increased compared to their parental cell lines (0.05±0.02 μM and 0.008±0.002 μM for PC9 and HCC827, respectively) (Fig 1A and 1B). Unlike the parental PC9 that has a spindle-like cell morphology, the PC9-GR subline showed a flat and hypertrophic morphology (Fig 1C). The HCC827-GR subline exhibited an elongated cell shape, while the parental HCC827 cells exhibited a typical epithelial morphology (Fig 1C). By using a highly sensitive genotyping assay, both PC9-GR and HCC827-GR sublines were found to harbor the primary EGFR mutation (exon19del E746-A750) and did not acquire any other EGFR mutations. In addition, there was no *MET* amplification in either of the gefitinib-resistant sublines. However, the expression of E-cadherin was significantly down-regulated, and the levels of N-cadherin and Vimentin were up-regulated in the PC9-GR and HCC827-GR sublines, indicating the occurrence of EMT in the gefitinib-resistant cell lines (Fig 1D). In parallel, the level of E-cadherin mRNA was reduced and levels of N-cadherin and Vimentin mRNA were elevated in the PC9-GR and HCC827-GR sublines (Fig 1E). The occurrence of EMT in the PC9-GR subline was further confirmed by analyzing the mRNA levels of more epithelial markers, including CLDN4, CLDN7, MUC1 and TJP3, and mesenchymal markers, Snail, Slug, Twist1, Twist2, ZEB1, ZEB2 and fibronectin. The mRNA levels of all the epithelial markers were down-regulated in the PC9-GR and HCC827-GR sublines, while the mRNA levels of the mesenchymal markers, Slug, ZEB1 and ZEB2, were up-regulated in both sublines; however, the expression of Twist1 was increased in the HCC827-GR subline (Fig 1F and 1G).

### Slug- and Snail-induced epithelial-to-mesenchymal transition promotes gefitinib resistance in the EGFR-mutant lung cancer cell lines

**Fig 2:**
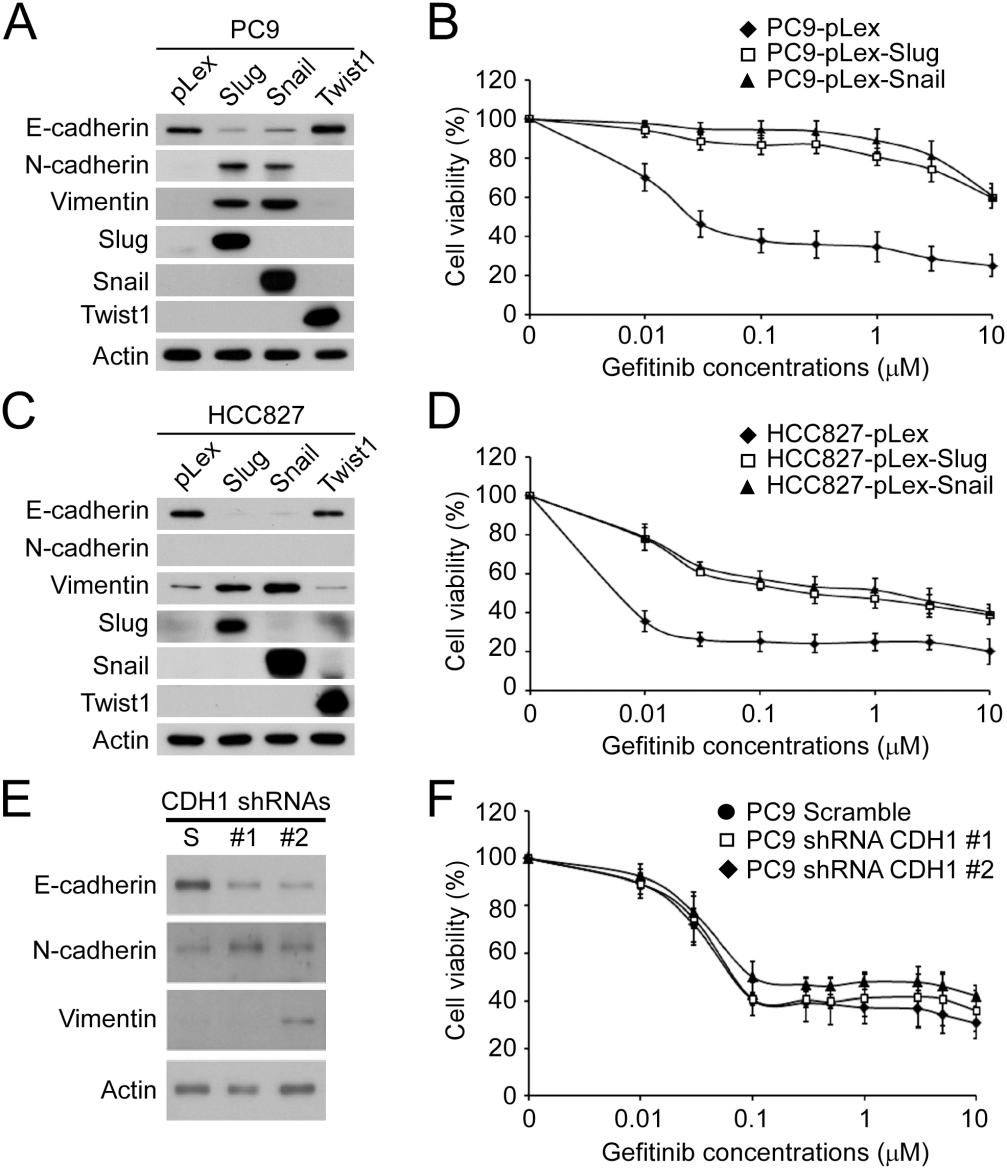
Snail- and Slug-induced EMT promoted drug resistance of parental PC9 and HCC827 cells. (A) The protein levels of EMT biomarkers and ectopically expressed Slug, Snail and Twist1 in the parental PC9 cells. (B) The pLex-, pLex-Slug- and pLex-Snail-expressing parental PC9 cells were treated with gefitinib at the indicated concentrations for 72 hours, and the relative cell viability was determined by MTT assay. (C) The protein levels of EMT biomarkers and the ectopically expressed Slug, Snail and Twist1 in the parental HCC827 cells. (D) The pLex-, pLex-Slug- and pLex-Snail-expressing parental HCC827 cells were treated with gefitinib at the indicated concentrations for 72 hours, and the relative cell viability was determined by MTT assay. (E) Endogenous E-cadherin (*CDH1*) was knocked down by two shRNA clones (#1 and #2). The protein levels of EMT biomarkers were examined by Western blot. (F) Stable clones of scramble shRNA-, shRNA #1- and shRNA #2-transduced PC9 cells were treated with gefitinib at the indicated concentrations for 72 hours, and the relative cell viability was determined by MTT assay.

Slug has been identified as an EMT inducer and contributes to gefitinib resistance in NSCLC [25]. In addition, Snail, the downstream molecule of hedgehog signaling pathway also contributes to TKI resistance in EGFR-TKI-resistant cells [8]. To demonstrate the effect of EMT on the TKI resistance of PC9 and HCC827 cells, strong transcriptional inducers of EMT, Slug, Snail and Twist1, were ectopically expressed in the parental PC9 and HCC827 cells by lentiviral transduction. Forced expression of Snail or Slug but not of Twist1 successfully induced EMT in the parental PC9 cells as shown by the decrease of E-cadherin expression and increase of N-cadherin and Vimentin expression (Fig 2A). The ectopic expression of Slug or Snail resulted in the gefitinib resistance of parental PC9 cells compared to the vector-only control cells (IC_50_ = 0.04±0.02 μM, 9.0±1.8 μM and 11.8±2.0 μM for the PC9-pLex, PC9-pLex-Slug and PC9-pLex-Snail cells, respectively) (Fig 2B). In the parental HCC827 cells, only the ectopic expression of Slug or Snail was capable of inducing EMT (Fig 2C). Similar effect of Slug- or Snail-induced EMT on gefitinib resistance was also observed in the parental HCC827 cells, where the forced expression of Slug or Snail increased the IC_50_ of gefitinib (IC_50_ = 0.019±0.007 μM, 1.4±0.6 μM and 2.4±0.2 μM for the HCC827-pLex, HCC827-pLex-Slug and HCC827-pLex-Snail cells, respectively) (Fig 2D). Since the expression of E-cadherin is the most reliable marker for EMT, we were interested in studying whether the loss of E-cadherin expression was sufficient to induce gefitinib resistance. For this purpose, we established stable derivates of PC9 cells by lentiviral delivery of scramble control shRNA or two E-cadherin shRNA clones. Knockdown of E-cadherin reduced the expression of E-cadherin and increased the expression N-cadherin, but the increase in vimentin expression could only be observed with the shRNA clone #2, suggesting the occurrence of EMT in the E-cadherin-knockdown PC9 cells (Fig 2E). However, the E-cadherin knockdown-induced EMT did not change the drug response of the PC9 cells to gefitinib (IC_50_ = 0.08±0.01 μM, 0.08±0.02 μM and 0.1±0.006 μM for the PC9-scramble, PC9-shRNA #1 and PC9-shRNA #2 cells, respectively) (Fig 2F).

### Recovery of gefitinib sensitivity and reverse EMT in the gefitinib-resistant cell lines after prolonged withdrawal of gefitinib

**Fig 3:**
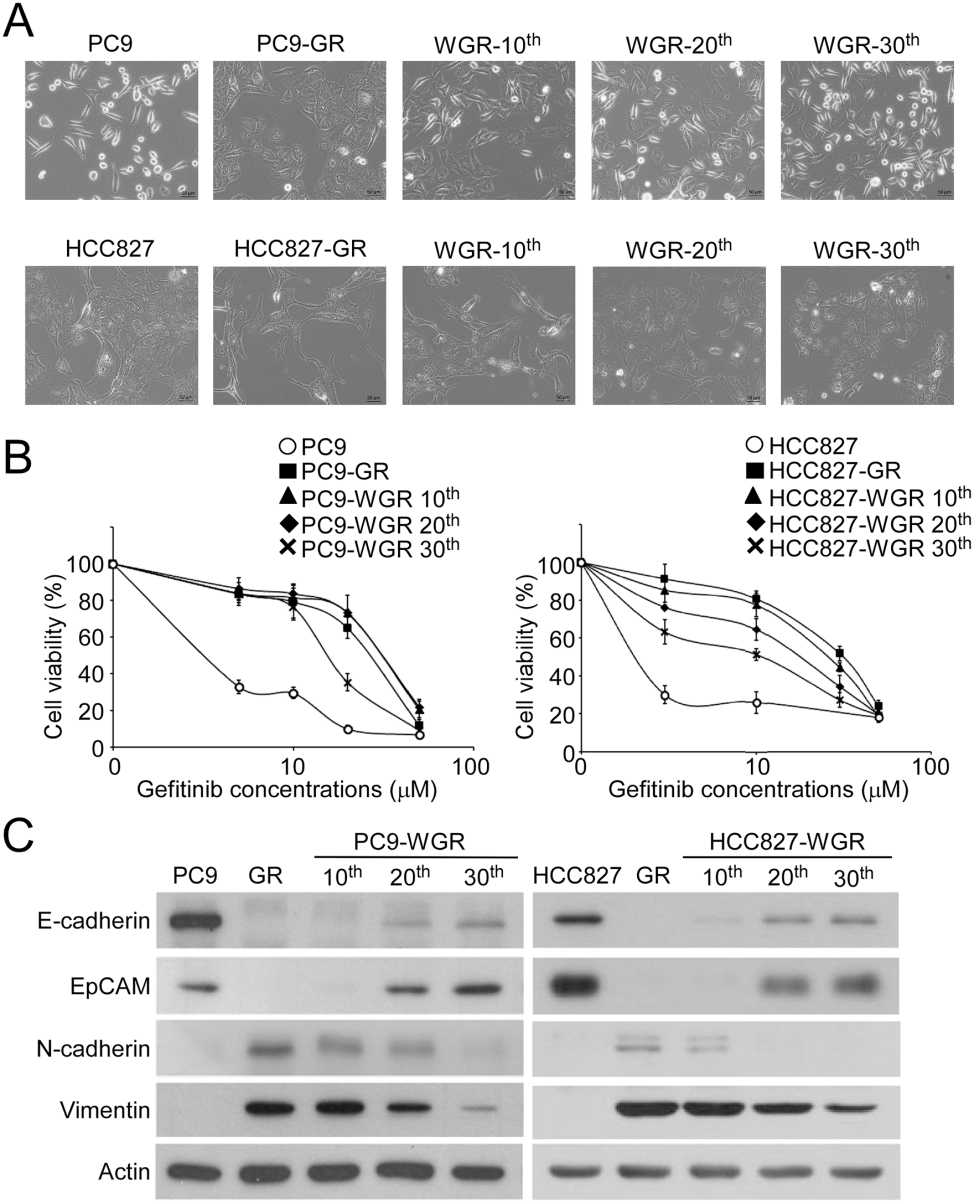
Prolonged gefitinib withdrawal sensitized the gefitinib-resistant cells and induced reverse EMT. (A) Bright-field microscopy images of the parental PC9/HCC827, gefitinib-resistant PC9 (PC9-GR)/HCC827 (HCC827-GR), and gefitinib-withdrawn PC9-GR (PC9-WRG)/HCC827-GR (HCC827-WGR) cells at different passages. (B) Parental, GR and WGR PC9 (left panel) and HCC827 cells (right panel) at different passages were treated with gefitinib at the indicated concentrations for 72 hours, and the relative cell viability was determined by MTT assay. (C) The protein levels of EMT biomarkers in the parental, GR and WGR PC9 (left panel) and HCC827 cells (right panel) at different passages were analyzed by Western blot.

Clinical observations showed that lung cancer patients with EGFR mutations who developed TKI-resistance and received chemotherapy as the second-line treatment remained responsive to the original TKIs as the third-line treatment. To demonstrate this possibility in vitro, gefitinib was removed from the culture medium (PC9-WGR and HCC827-WGR) of the PC9-GR and HCC827-GR sublines, which were maintained in gefitinib-containing culture medium. The cell morphology, gefitinib response and EMT status of the parental, GR and WGR PC9 and HCC827 sublines were monitored every 10 passages. Bright-field microscopy images showed that the cell morphology of the PC9-WGR subline gradually reverted to the spindle-like shape of the parental PC9 cells after 30 passages (Fig 3A, upper panels). A similar morphological change was also observed in the HCC827-WGR subline (Fig 3A, lower panels). Importantly, the PC9-WGR and HCC827-WGR sublines became more sensitive to gefitinib in a passage-dependent manner (Fig 3B and Table 1). Accompanied with the increase in TKI sensitivity in the PC9-WGR and HCC827-WGR sublines, the expression of epithelial markers E-cadherin and EpCAM was up-regulated and the expression of mesenchymal markers N-cadherin and Vimentin was down-regulated in the PC9-WGR and HCC827-WGR sublines (Fig 3C). These results suggested that the regain of gefitinib sensitivity in the gefitinib-resistant lung cancer cells after prolonged gefitinib withdrawal is associated with the reversal of EMT.

**Table 1:** The IC_50_ values of the PC9-WGR and HCC827-WGR cells decreased after prolonged withdrawal of gefitinib.

Cell lines	parental cells	resistant cells (GR^a^)	passages after gefitinib withdrawal (WGR^b^)		
			10th	20th	30th
PC9	0.07±0.006	33.6±1.9	33.3±1.4	28.4±3.5	16.4±0.7*
HCC827	0.01±0.002	30.9±1.1	25.7±1.4	23.0±2.1*	15.9±2.3*

The unit for all IC_50_ values is μM.

* *p*<0.05, compared with the gefitinib-resistant sublines

^a^ GR, gefitinib resistant

^b^ WGR, gefitinib withdrawal from the culture medium of gefitinib-resistant sublines

### Blockage of reverse EMT leads to the increase in gefitinib resistance

**Fig 4:**
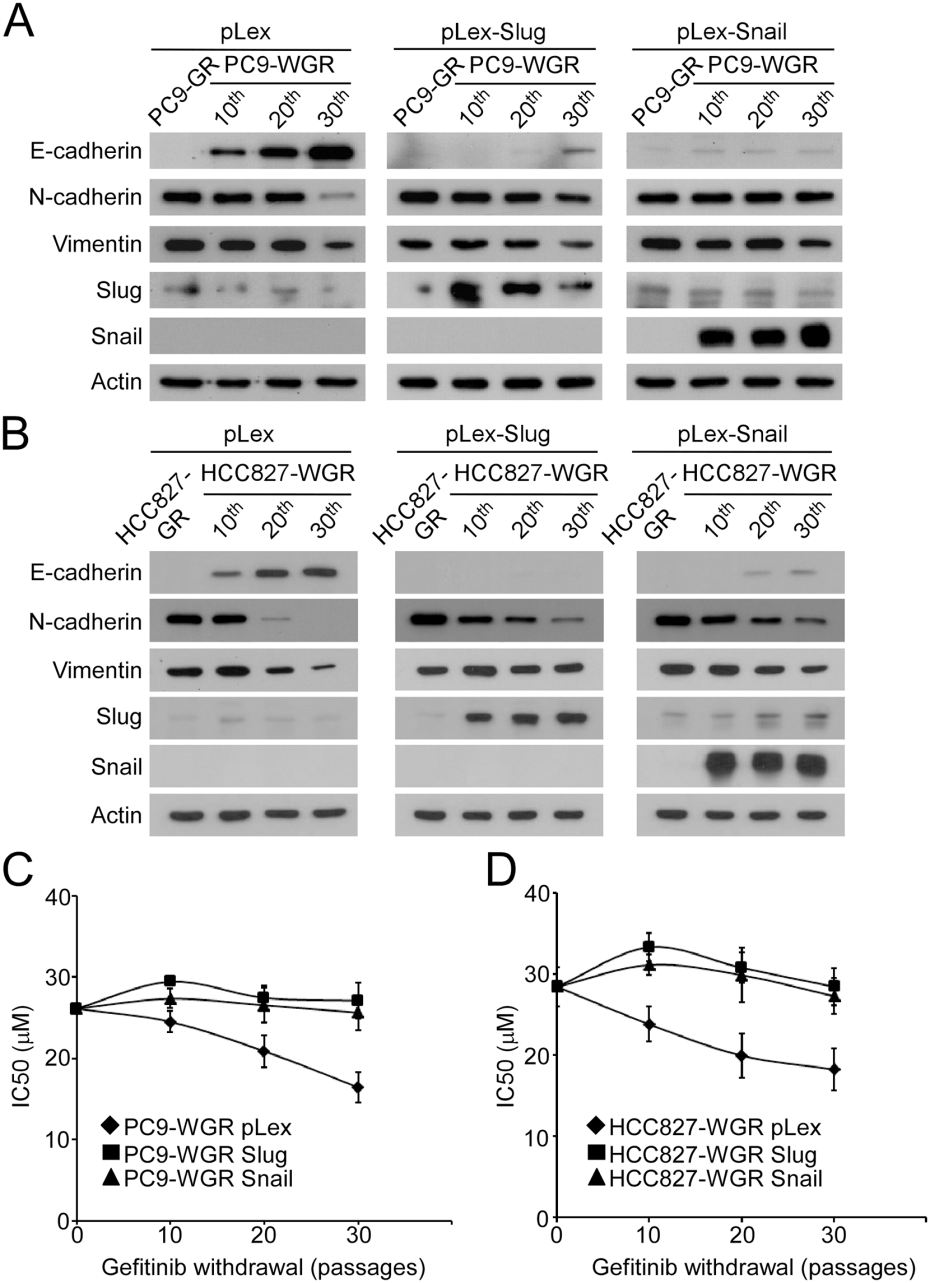
Blockage of gefitinib withdrawal induced reverse EMT, and the forced expression of Snail and Slug inhibited the regain of gefitinib sensitivity. (A) The vectors pLex, pLex-Slug and pLex-Snail were introduced into the PC9-GR and PC9-WGR cells at different passages. The protein levels of EMT biomarkers were examined by Western blot. (B) The vectors pLex, pLex-Slug and pLex-Snail were introduced into the HCC827-GR and HCC827-WGR cells at different passages. The protein levels of EMT biomarkers were examined by Western blot. (C) Parental, GR and WGR PC9 cells at different passages were treated with gefitinib at the indicated concentrations for 72 hours, and the relative cell viability was determined by MTT assay. The IC_50_ values at each passage after gefitinib withdrawal were determined and plotted. (D) Parental, GR and WGR HCC827 cells at different passages were treated with gefitinib at the indicated concentrations for 72 hours, and the relative cell viability was determined by MTT assay. The IC_50_ values at each passage after gefitinib withdrawal were determined and plotted.

To investigate whether reverse EMT is the cause of the regain of gefitinib sensitivity, Slug or Snail was ectopically expressed in the PC9-GR and PC9-WGR sublines. In the vector-only control group, reverse EMT occurred after prolonged withdrawal of gefitinib from the culture medium. Ectopic expression of Slug or Snail diminished the increase in E-cadherin and decrease in N-cadherin and Vimentin in the PC9-WGR subline (Fig 4A). Similarly, ectopic expression of Slug or Snail weakened reverse EMT in the HCC827-WGR subline (Fig 4B). We further monitored the drug response of the PC9-WGR and HCC827-WGR sublines to gefitinib for 30 passages. As shown in Fig 4C, the ectopic expression of Slug or Snail resulted in a constant IC_50_ of gefitinib in the PC9-WGR subline, while the IC_50_ of gefitinib was significantly reduced in the vector-only control group. In parallel, the forced expression of Slug or Snail blocked the regain of gefitinib sensitivity in the HCC827-WGR subline. Together, these results demonstrated that the regain of gefitinib sensitivity after prolonged withdrawal of gefitinib was mediated by reverse EMT in the TKI-resistant lung cancer cells.

## Discussion

Since the discovery of TKIs, targeted therapy has become the most effective way to manage lung cancer. Despite the initial response of patients to TKIs, most of the patients still experience tumor recurrence due to acquired resistance to TKI and receive chemotherapy as their second-line treatment [26]. However, clinical and pre-clinical studies have revealed that TKI resistance is a transient event and may be reversed by the long-term culture of TKI-resistant lung cancer cells in TKI-free medium or by treatment with inhibitors of insulin-like growth factor 1 receptor or chromatin-modifying agents [7]. In the current study, we demonstrated that EMT is one of the major causes of gefitinib resistance in two NSCLC cell lines. In addition, the regain of TKI sensitivity could be mediated by reversal of EMT, since the blockage of reverse EMT by the constitutive expression of EMT inducers, Snail and Slug, can inhibit this effect. Our findings provide an important insight showing that EMT inhibitors may sensitize lung cancer cells to TKIs and prolong the efficacy of TKIs in lung cancer patients who carry EGFR mutations.

In addition to findings in vitro, EMT has been linked to the poor clinical outcomes of lung cancer patients [27, 28], especially those who received TKI therapy [29, 30]. In our data, gefitinib-resistant PC9 and HCC827 cells transited to a mesenchymal phenotype, suggesting the occurrence of EMT. However, we cannot exclude the possibility that this phenomenon is caused by the clonal selection of originally existing mesenchymal-type cells in the heterogeneous cell culture. Thus, the question of whether EMT is the cause or consequence of drug resistance remains. A previous study has indicated that cisplatin treatment induced EMT and resulted in the subsequent resistance to gefitinib [31]. Thus, although EMT might be the consequence of chemotherapy, EMT can also be the cause of TKI resistance. Consistently, the initiation of EMT by introducing EMT inducers, Snail or Slug, increased the resistance of PC9 and HCC827 cells to gefitinib and blocked the regain of TKI sensitivity mediated by long-term culture in gefitinib-free medium, suggesting that EMT leads drug resistance of lung cancer cells. Interestingly, we noticed that the gefitinib-resistant PC9 and HCC827 cells were not resistant to cisplatin, indicating that other mechanisms may be involved in cisplatin resistance. In addition, ZEB1 and ZEB2 were highly up-regulated in the PC9-GR and HCC827-GR cells in our study. Previous study has indicated that ZEB1 contributed to TKI-resistance in NSCLC [21]. Further experiments for the roles of ZEB1 and ZEB2 in TKI resistance and TKI holiday are required.

Dose-escalation protocols have been widely used to establish TKI-resistant NSCLC cell lines with EGFR mutations. However, different experimental procedures between the protocols may lead to phenotypic or genotypic variations of the TKI resistant cells. For example, continuous exposure of PC9 cells to increasing concentrations of gefitinib results in the frequent T790M mutation, but intermittent treatment did not [32]. Importantly, some studies indicated that long-term withdrawal of gifitinib from the culture medium of the gefitinib-resistant PC9 cells with EGFR T790M mutation (no *MET* amplification) does not alter the gefitinib-resistant phenotype of the cells [32, 33], but the gefitinib-resistant PC9 cells without EGFR T790M mutation or *MET* amplification regain the sensitivity to gefitinib [32], which is consistent with our findings in this study. It is worthy to note that different TKI-resistant cell lines with T790M mutation may have different responses to the long-term TKI withdrawal [24]. However, the EMT status of the TKI-resistant cells was not examined in these studies. In our study, the PC9-GR cells did not harbor T790M mutation and *MET* amplification, but displayed EMT phenotypes. Similar observation was also reported from other groups [25, 34]. Our data indicated that constitutive maintenance of EMT prevented the TKI withdrawal-mediated regain of TKI sensitivity, suggesting the EMT could be an important mechanism for TKI resistance in NSCLC without T790M mutation. However, previous study reported that inhibition of EMT was not sufficient to prevent acquired gefitinib resistance because of an increased emergence of the EGFR T790M mutation [35]. Due to the complexity of EMT and genetic mutations of EGFR associated with TKI resistance in heterogeneous subpopulations of a tumor, the roles of EMT and EGFR mutations in TKI resistance should be further investigated.

Unlike Snail and Slug, the overexpression of Twist1 did not induce EMT under our experimental conditions, although these three transcription factors inhibit the expression of E-cadherin [27, 36]. It has been shown that Twist1 was not up-regulated in cisplatin-resistant lung cancer cells [37, 38]. Due to the pro-survival function of Twist1, overexpression of Twist1 potentiated drug resistance to chemotherapy agents, but whether this resistance is associated with its ability to induce EMT remains to be resolved [36]. Because the function of Twist1 in EMT and drug resistance is highly dependent on the cell type and tissue context [39], further study is required for the elucidation of Twist1-mediated molecular mechanisms.

Many environmental factors and intracellular proteins that can promote EMT have been identified [40]. However, intrinsic EMT inhibitors are rarely identified [41]. Several microRNAs have been shown to inhibit EMT by suppressing the expression of EMT inducers [42, 43]. MicroRNA-147 inhibits EMT and reverses the drug resistance of lung cancer cells to TKIs [44]. Several synthetic and natural compounds exert anti-EMT effects on lung cancer cells [45, 46]. Dasatinib, an ABL/Src kinase inhibitor, is capable of inhibiting TGF-β-induced EMT in lung cancer cells [20]. Our study provides fundamental evidence showing that EMT is one of the important mechanisms of TKI resistance, and reverse EMT results in the regain of gefitinib sensitivity. The development of novel small molecules targeting the EMT process may contribute to the efficacy and reuse of TKIs in NSCLC patients.
